# Clinical Impact of Ketogenic Diet

**DOI:** 10.3390/nu18020245

**Published:** 2026-01-13

**Authors:** Valentina Guarnotta

**Affiliations:** Unit of Endocrinology, Department of Health Promotion, Mother and Child Care, Internal Medicine and Medical Specialties, Policlinico Paolo Giaccone, Università Degli Studi di Palermo, 90127 Palermo, Italy; valentina.guarnotta@unipa.it; Tel.: +39-3807049652

During the last century, the ketogenic diet (KD) has gradually shifted from a specialized dietary therapy used almost exclusively in pediatric epilepsy to a metabolic intervention explored across several areas of clinical medicine. This evolution reflects increasing recognition of ketosis as a systemic metabolic state capable of influencing neurological function, inflammatory signaling, endocrine regulation, and energy balance. The articles included in this Special Issue, Clinical Impact of Ketogenic Diet, illustrate both the therapeutic opportunities offered by ketogenic strategies and the challenges associated with their clinical implementation. Neurology remains the field with the most robust evidence supporting KD use [1]. The review by Na et al. (contribution 1) provides a framework for integrating ketogenic therapy into the management of genetically determined drug-resistant epilepsy, emphasizing the limitations of standardized dietary prescriptions and the need for individualized approaches based on genetic and metabolic profiles. Available protocols, including the classic ketogenic diet (cKD), modified Atkins diet (MAD), medium-chain triglyceride diet (MCTD), and low glycemic index treatment (LGIT), enable tailoring of therapy to clinical needs and tolerability.

In conditions such as GLUT1 deficiency syndrome and pyruvate dehydrogenase complex deficiency, ketogenic therapy remains a first-line intervention. In other genetic epilepsies, including those associated with SCN1A, KCNQ2, and CDKL5, responses are heterogeneous but clinically meaningful, with less restrictive diets often preferable when adherence to cKD is limited. Equally important is recognizing contraindicated settings, particularly fatty acid oxidation disorders and mitochondrial diseases, which require careful patient selection and close metabolic monitoring.

Interest in ketogenic strategies has extended into psychiatry, where altered cerebral energy metabolism and neuroinflammation are increasingly implicated in disease mechanisms [2]. Chrysafi et al. (contribution 2) examined ketogenic approaches in depression, anxiety, schizophrenia, stress-related disorders, and bipolar disorder, encompassing dietary models ranging from the classic KD to modified low-carbohydrate/high-fat regimens, Atkins variants, MCT diets, and ketone supplementation. Preclinical studies show favorable effects on mood and stress regulation. However, clinical evidence remains inconsistent, largely derived from small, short-term, uncontrolled studies. More promising signals have emerged in psychotic disorders and bipolar illness, yet definitive conclusions await well-powered trials with standardized outcomes.

Translational insights are further illustrated by the study of Allan et al. (contribution 3) in children with autism spectrum disorder. Dietary ketosis was associated with changes in gut microbiota composition, reductions in inflammatory cytokines, and modulation of circulating microRNAs linked to neurotrophic pathways, supporting the involvement of the gut–immune–brain axis in mediating ketogenic effects beyond behavioral observations alone.

Metabolic applications represent another active area of investigation [3]. Emanuele et al. (contribution 4) reviewed trials of very-low-calorie ketogenic diets in metabolic dysfunction–associated steatotic liver disease (MASLD), documenting short-term reductions in hepatic fat content and improvements in insulin sensitivity. Given the limited pharmacological options for MASLD, these findings are clinically relevant, though longer studies remain necessary to assess cardiovascular outcomes and lipid metabolism.

Systemic inflammation constitutes a shared pathophysiological feature across obesity-related disorders. In this context, Rondanelli et al. (contribution 5) conducted a meta-analysis of randomized trials evaluating inflammatory biomarkers after ketogenic interventions, including classic KD, VLCKD, and modified protocols, compared with standard diets. Most studies reported reductions in C-reactive protein (CRP), while effects on IL-6 were less consistent. These findings align with experimental evidence implicating β-hydroxybutyrate-mediated suppression of NLRP3 inflammasome activity, although the modest size of available trials limits definitive conclusions.

Despite these biological benefits, real-world translation of KD is constrained by adherence challenges. García-Gorrita et al. (contribution 6) assessed a personalized ketogenic program in a large clinical cohort, reporting rapid reductions in body weight and fat mass over three months with preservation of lean tissue. However, declining adherence and partial weight regain during follow-up underscored the importance of behavioral support and sustainable dietary frameworks for maintaining long-term benefit.

Safety considerations remain essential, particularly in populations with comorbid disease [4]. Verde et al. (contribution 7) evaluated Phase 1 Very Low Energy Ketogenic Therapy (VLEKT) in patients with obesity and mild renal impairment, demonstrating improvements in weight, metabolic parameters, and lipid profiles without renal deterioration. Modest increases in eGFR were observed in those with baseline impairment, supporting short-term safety under medical supervision while highlighting the need for longer-term evaluation.

Experimental findings further emphasize biological variability in ketogenic responses [5]. Sprankle et al. (contribution 8) demonstrated sex- and age-dependent metabolic effects of KD in murine models, with adverse lipid accumulation and glucose intolerance more pronounced in older males, while transient motor improvements were limited to young females. These results underscore the importance of stratification in both preclinical research and clinical translation.

Collectively, the contributions to this Special Issue shows KD as a therapeutic tool with demonstrated benefits in selected settings but limited generalizability without personalized assessment. Progress will depend on improved patient stratification using genetic, metabolic, and inflammatory profiling, alongside behavioral strategies supporting dietary persistence. Multidisciplinary care integrating nutritional supervision, medical oversight, and psychological support appears fundamental to sustaining efficacy.

Viewed in this framework, ketogenic therapy should not be regarded as a standardized dietary template but as a targeted medical intervention whose effectiveness depends on appropriate indication, careful monitoring, and long-term patient engagement. The studies presented here provide valuable evidence to refine this clinical approach and guide future research aimed at defining the precise role of ketogenic diets within evidence-based metabolic therapy.

## Abbreviations

The following abbreviations are used in this manuscript:

KD	Ketogenic diet
cKD	Classic ketogenic diet
MAD	Modified Atkins diet
MTCD	Medium-chain triglyceride diet
LGIT	Low-glycemic index treatment
GLUT1	Glucose transporter type 1
SCN1A	Alpha-1 subunit of the voltage-gated sodium channel
KCNQ2	Potassium voltage-gated channel subfamily Q member 2
CDKL5	Cyclin dependent Kinase-like 5
MASLD	Metabolic-dysfunction-associated steatotic liver disease
CRP	C-reactive protein
NLRP3	NLR family pyrin domain containing 3
VLEKT	Very Low Energy Ketogenic Therapy
